# 

**Published:** 1893-02-11

**Authors:** 


**Notices of Births, Deaths, and Marriages,** are inserted in this column at a charge of 2s. 6d. for an announcement not exceeding four lines.

### NOTICE TO CORRESPONDENTS.

All MS., Letters, Books for Review, and other matters intended for the Editor should be addressed The Editor, The Lodge, Porchester Square, London, W.

The Editor cannot undertake to return rejected M.S., even when accompanied by stamped directed envelope.

**Notice to Advertisers and Subscribers.**

All Advertisements, Orders for Copies of The Hospital, and Business Communications should be addressed to The Publisher, *not to the Editor*, The Hospital, 140, Strand, W.C.

The Cost of Subscription, post free, is as follows:—Per week, 2½d.; per quarter, 2s. 6d.; per half-year, 5s.; per annum, 8s. 6d. Foreign:—Per annum, 10s. 8d.; payable, in all cases, in advance.

**"Burdett's Hospital Annual," 1893.**

Fourth year of publication. 700 pp. Boards, gilt lettering. A most complete guide to British, Colonial, Indian, and American Hospitals, Dispensaries, Nursing and Convalescent Institutions, and Asylums. Price 5s. Published by The Scientific Press (Limited), 140, Strand, W.C.

**NOTICE.—The Index for Vol. XII. (ending September 1892) is on sale at the Office of this Journal, price 2½d., post free.**

### SCRAPS AND GLEANINGS.

It is computed that the sale of pills in the United Kingdom is at the rate of 200 000,000 annually.

The Ventnor Royal National Hospital for Consumption has received a donation of £400 from Baron Hirsch.

Arrangements have been made at Retford for a course of six free weekly lectures on Health and Sick Nursing.

Over 300 temperance sermons will be preached at 190 churches in the diocese of Liverpool on "Temperance" Sunday.

Electric railways are now to be supplied to Japan, which country can already boast of its telephones and electric light.

The largest number of medical decrees taken at the same time was at Edinburgh last year, when 202 M. B.'s were presented and 60 M.D.'s.

An ingenious watchmaker at Geneva has lately applied the phonograph to his clocks, thus causing the hours to be spoken instead of chimed.

The London Homœopathic Hospital has just received 50 guineas from Mrs. E. B. Stevenson, who has won this sum through a competition prize.

Queen Liliokalani, the sovereign of the Sandwich Islands, has become a teetotaler, and is advocating the cause to her country-people.

Chelmsford is again mooting the question of providing itself with an infectious diseases hospital. A committee is formed to promote the scheme.

The Subscription Ball, which took place in the Holborn Town Hall in aid of the Royal Free Hospital, has contributed £134 to the funds of that institution.

The mortality from diphtheria last year in Hungary was quite appalling. In 25 counties 23,070 children were attacked, and 9,137 died in 12 months.

The first meeting of the Royal Commission for inquiring into the question of old-age pensions was held on Wednesday, the 1st inst., in the House of Lords.

The analysis of restaurant coffee, as recently defined by a Parisian journal, is a mixture of horse liver roasted in an oven, black walnut sawdust, and caramel.

It is reported from China that that country imports 12,000,000 fans annually, and, therefore, the entire manufacture of those articles is not centred in Birmingham.

It is asserted that a royalty approaching 1,200,000 dollars, accruing from the sale of Moody and Sankey's hymns, has been awarded to charitable purposes in America.

The Princess of Wales and her two daughters are contributing specimens of their leather work to the Chicago Exhibition. The proceeds of the sale will be devoted to charitable purposes.

Workhouse Guardians are now empowered to visit workhouses at any time they choose; previously no individual Guardian could thus visit unless specially authorized by the Board of Guardians.

The Dowager Duchess of Newcastle has purchased St. Raphael's Hospital, Worthing, and it will shortly be opened as a convalescent home under the direction of the Bermondsey Sisters of Mercy.

The streets at Cannes are the most carefully cleaned of any in Europe. A "Street Cleaning Brigade" twice daily sweep the town with long handled brooms, afterwards laying the dust with watering cans.

Mr. R. A. Scott, of Dumfries, has presented the Dumfries Galloway Royal Infirmary with an ambulance waggon of the most approved and recent construction for the conveyance of surgical cases to the house.

Gout can no longer be said to confine itself to the aged; indeed, its most favoured prey seems now to be the younger people. We have just read the record of a typical case occurring in a child of only eleven years of age.

Mr. Owen Breden is to be congratulated on his organisation of the late entertainment at the Brompton Hospital, both the dramatic and musical portion of the programme being greatly appreciated by the audience.

Scientists have measured the thickness of the envelope of soapy water enclosing the air of a bubble when it becomes so thin as to produce rainbow tints, the violet wave of light being computed at 1·60,000 of an inch.

A special envoy representing the Chicago Fair Committee will leave shortly for England in order to invite the Prince of Wales to visit the Exhibition. Their invitation is inscribed on elaborately embossed parchment.

The Manchester Hospitals Work Society supplies now 15 hospitals with garments for the use of the poor while they are inmates. The number of these articles which have been thus distributed amounted to 1,324 last year.

A new departure in the treatment of the lame has just been effected through Mr. E. O'Connor's invention of an "extension shoe." This shoe, expanding from two to ten inches, equalises to all appearances the length of the limbs.

Provost Reid is again a benefactor to the Forfar Infirmary. This time he proposes to erect a convalescent ward for all the patients in the institution who may be able to take advantage of it. This hall will probably be 23 by 30 feet in size.

Pleuro pneumonia has been stamped out by the Bureau of Animal Industry in the United States. America is the first nation, extensively infected, which has extirpated the disease. This took five years to effect, at the cost of 1,500,000 dollars.

Alum has been found to be the best clarifier and purifier of water, the old system of filtration being proved useless for removing micro-organisms. Powdered alum thrown in the drinking water and left to stand for 24 hours is the best substitute for a filter.

Dr. R. M. Phelps, of the Minnesota Hospital for the Insane, has proved the value of strychnine in inebrity. Nitrate of strychnia, taken in certain specified doses, firstly impairs the appetite for liquid, and later this medicine becomes an incompatible or sickening agent.

For Student of Medicine and Medical Appointments see page VIII. and IX. overleaf.

### THE STUDENT OF MEDICINE.

[All communications for this Supplement should be addressed to THE EDITOR of THE HOSPITAL, at 140, Strand, with the words, "Students' Column," written in the left hand top corner of the envelope.]

### APPOINTMENTS.

Anderson, James, M.D.Aberd., F.R.C.P.Lond., appointed Physician to Out-patients, National Hospital for the Paralysed and Epileptic, Queen Square, Blooomsbury. Bennett, C.J., M.R.C.S., appointed Consulting Surgeon to the Cheltenham General Hospital. Brown, Haydn, L.R.C.P., L.R.C.S.Edin., L.F.P.S. Glas., appointed Surgeon to the Hospital of St. Peter Port, Guernsey. Burton Fanning, F. W. M.B.Camb., M.R.C.P.Lond., M.R.C.S., appointed Pathologist and Curator of the Museum at the Norfolk and Norwich Hospital. Burr, W. T., M.B.T.C.D., M.Ch., appointed Medical Officer for the Gamlingay District of the Caxton and Arrington Union, and Medical Officer for the Third District of the St. Neots Union. Calcott, G. W. B., M.R.C.S., appointed Medical Officer for the Oundle Sanitary District of the Oundle Union. Carruthers, J. F., M.B., C.M.Edin., appointed Medical Officer for the Renesby District of the Horncastle Union. Clarke, Mr. John H., appointed Medical Officer of the Hanna District of the Ellesmere Union. Dennis, Ffolliott Reginald, L.R.C.P., L.R.C.S.Edin., appointed Medical Officer for the Trant District of the Ticehurst Union. Douglas, N.G., L.R.C.P., L.R.C.S. Edin., appointed Medical Officer for the Scarborough Western District of the Scarborough Union. Gilpin, Robert Harrison, M.R.C.S., L.R.C.P.Lond., appointed Medical Officer of Health for the Gloucestershire Parishes of the Evesham Rural Sanitary Authority. Grigor, Macadam, L.R.C.P., L.R.C.S.Edin., appointed Medical Officer and Public Vaccinator for the No. 2 District of the Parish of Battersea. Horrocks, Herbert, M.D. Lond..M.R.C.S., L.R.C.P., appointed Junior House Surgeon to the North-Eastern Hospital for Children. Jackson, Mr. H., V., appointed Medical Officer for the Cooper's Lane District of the Edmonton Union. Kempe, Arthur W., M.D.Brux., M.R.C.P. Edin, M.R.C.S.Eng., reappointed Medical Officer of Health to the Exeter Port Sanitary Authority. Kempster, Wm. Henry, M.B.Durh., M.R.C.S.Eng., appointed Medical Officer and Public Vaccinator for the No. 1 District of the Parish of Battersea. Kilner, C. S., M.B., C.M. Edin., D.P.H.Camb., appointed Senior Medical Officer of the Suffolk General Hospital. Livesay, A.W.B., M.B., C.M.Edin., appointed Assistant House Surgeon to the Derbyshire Royal Infirmary, Derby. Lovett, Dr. G.V., appointed Senior Resident Medical Officer at the Public Hospital, Kingston, Jamaica. McKinnon, Archibald A., M.R.C.S.Eng., L.R.C.P. Lond., appointed House Surgeon to the Central London Ophthalmic Hospital, Gray's Inn Road, W.C. Mayner, Dr., appointed Junior Resident Medical Officer at the Public Hospital, Kingston, Jamaca. Metcalfe, G.E.G., L.R.C.P., L.R.C.S.Edin., appointed Medical Officer of the Third District of the Tonbridge Union. Millar, Dr. appointed Medical Officer to the Lossiemouth Oddfellows. Norton, Everitt C., L.R.C.P.Lond., M.R.C.S.Eng., appointed Resident Medical Officer of the City Road Workhouse of the Holborn Union. Pearce, Mr. F. E., appointed Medical Officer for the Ripley District of the Guildford Union. Pollard, F., M.D.Lond., appointed Medical Officer of the School of the Westminster Union. Price, R. G., M.D., L.R.C.P., appointed Certifying Factory Surgeon, Carmarthen. Roberts, C.H., L.R.C.PLond., M.R.C.S., appointed Honorary Visiting Medical Officer to the Hostel of St. Luke. Serachan. Wm. Henry Williams, L.R.C.P.Lond., M.R.C.S.Eng., appointed Senior Medical Officer at the Public Hospital, Kingston, Jamaca. Southey, Albert James, M.R.C.S.Eng., L.S.A., reappointed Medical Officer of Health for the Eton Urban Sanitary Authority.

### The Hospital Advertiser.

### MEDICAL APPOINTMENTS.

The following vacancies are announced this week, the amount of salary in each case being given after the name of the institution:

**Resident Medical Officers.**

Boscombe Hospital, Bournemouth. House Surgeon. Salary £60 per annum, with board and residence. Unmarried. Applications to Honorary Secretary by February 11th.

Chichester Infirmary, Chichester. House Surgeon. Salary £80 per annum, with board, lodging; and washing. Applications to E. E. Street, Secretary, by March 1st.

London Lock Hospital. House Surgeon. Member of the Royal College of Surgeons. Salary, £100 per annum, with board, lodging, and washing. No private practice allowed. Applications to Geo. Gilbert, Secretary, by February 20th.

Dundee Royal Infirmary. Medical Superintendent. Applications to the Secretary, D. Gordon Stewart, Solicitor, Dundee, by February 8th.

Stockport Infirmary. Junior Assistant House Surgeon. Appointment for six months, with board and residence, and honorarium of £10 at the end of six months' satisfactory service. Applications to the Secretary by February 6th.

**Non-Resident Medical Officers.**

Borough of Royal Leamington Spa. Medical Officer for Police Force. Average emoluments £35 per annum. Applications, marked "Applications for the Police Surgeon," to Mr. H. Consett Passman, Town Clerk, Town Hall, Leamington, by February 13th.

Guy's Hospital, S.E. Assistant Dental Surgeon. Must possess L.D.S. Eng. qualification. Applications to the Dean by February 4th.

National Hospital for Diseases of the Heart, &c., 32, Soho Square, W. Physician to Out-patients; must be a Fellow or Member of the College of Physicians of London and a graduate in Medicine of a University. Applications to the Secretary by February 9th.

Parish of Glenelg. Medical Officer for the Northern Division. Salary £95 per annum, with free house and garden. Applications to D. M. Lure, Inspector of the Poor, Glenelg, by February 21st.

Rochdale Union. District Medical Officer. Salary £40 per annum, exclusive of extra medical fees. Applications to Mr. R. A. Leach, Union Clerk, Union Offices, Townhead, Rochdale, by February 6th.

St. Mark's Hospital for Fistula and other Diseases of the Rectum, City Road, E.C. Anæsthetist. Honorarium £50 per annum. Applications to the Secretary by February 25th.

University College of South Wales and Monmouthshire, Cardiff. A Professor of Anatomy, and a Professor of Physiology. Stipend in each case £350 per annum. Applications to Ivor James, Registrar, by February 10th.

University of Edinburgh. Additional Examiner in Chemistry. Salary £75 per annum, and £10 allowed for travelling and other expenses if not resident in Edinburgh or neighbourhood. Applications to M. C. Taylor, Secretary, by February 6th.

University of Edinburgh. Additional Examiner in Public Health. Salary £40 per annum, and £10 allowed for travelling and other expenses if not resident in Edinburgh or neighbourhood. Applications to M. C. Taylor, Secretary, by February 6th.

University of Edinburgh. Additional Examiner in Botany. Salary £75 per annum, and £10 allowed for travelling and other expenses if not resident in Edinburgh or neighbourhood. Applications to M. C. Taylor, Secretary, by February 6th.

West End Hospital for Diseases of the Nervous System, Paralysis, and Epilepsy, 73, Welbeck Street, W. Anæsthetist. Applications to A. Bedborough, Secretary, by February 7th.

